# Association Between Stimulant Medication Use in Attention-Deficit/Hyperactivity Disorder (ADHD) and the Increased Risk of Upper Respiratory Tract Infections: A Retrospective Study

**DOI:** 10.7759/cureus.90386

**Published:** 2025-08-18

**Authors:** Kypros J Dereschuk, Patrick S Chen, Eduardo D Espiridion

**Affiliations:** 1 Psychiatry, Drexel University College of Medicine, Philadelphia, USA; 2 Psychiatry and Behavioral Sciences, Drexel University College of Medicine, Philadelphia, USA

**Keywords:** attention deficit hyperactivity disorder (adhd), children, pediatrics, stimulant medications, upper respiratory tract infection

## Abstract

Introduction

Attention-deficit/hyperactivity disorder (ADHD) is a common neurodevelopmental condition that can persist from childhood into adulthood. Stimulant medications such as methylphenidate and amphetamine derivatives are the mainstay of treatment, yet their potential immunomodulatory effects remain unclear. While most upper respiratory tract infections (URTIs) are benign and self-limiting, some may result in healthcare visits and lost productivity. Proposed mechanisms linking stimulant use to infection risk include sympathetic nervous system activation, hypothalamic-pituitary-adrenal axis modulation, stimulant-induced insomnia, and reduced salivary flow - all of which may impair mucosal immunity. This study aimed to evaluate whether stimulant medication use in ADHD is associated with increased risk of URTIs using a large real-world dataset.

Methods

We performed a retrospective cohort study using the TriNetX research network, which aggregates de-identified electronic health records from 149 healthcare organizations worldwide (>170 million patients). ADHD patients were identified by International Classification of Diseases, 10th Revision (ICD-10) codes F90.0-F90.2. Two cohorts were defined: ADHD patients without stimulant prescriptions (control; n = 1,798,001) and ADHD patients prescribed stimulants (medication cohort; n = 1,099,756; amphetamine, dextroamphetamine, lisdexamfetamine, methylphenidate, modafinil, or dexmethylphenidate). We did not include antidepressants and antipsychotics in the cohorts or in their comparison. The outcome was a diagnosis of URTI (ICD-10 J00-J06). Risk estimates, Kaplan-Meier survival analysis, log-rank test, and Cox proportional hazards modeling were performed within TriNetX, with significance set at p < 0.05.

Results

A total of 2,897,757 ADHD patients were included (mean age 21.2 ± 15 years; 42.1% female). URTI incidence was higher in the medication cohort (31.2%, n = 343,385) than in controls (28.5%, n = 512,849). Stimulant exposure was associated with increased URTI risk: risk difference 0.027 (95% confidence interval (CI): 0.026-0.028; p < 0.001), risk ratio 1.095 (95% CI: 1.091-1.099), and odds ratio 1.138 (95% CI: 1.132-1.144). Kaplan-Meier analysis demonstrated significantly lower URTI-free survival in medicated patients (log-rank χ² = 2285.0; p < 0.001). The hazard ratio for URTI in the medicated cohort was 1.111 (95% CI: 1.106-1.116; p < 0.001). Median survival without URTI was 3757 days in controls versus 3176 days in the medicated group.

Discussion

Stimulant-treated ADHD patients exhibited an 11% higher relative risk of URTI compared with unmedicated patients. Some possible mechanisms may include immune suppression via catecholamine-mediated shifts in cytokine profiles, sleep disruption leading to impaired host defenses, and medication-induced xerostomia, reducing mucosal protection. Our findings align with prior clinical and epidemiologic studies reporting higher infection rates in ADHD populations, although prior results have been mixed.

Conclusion

In this large multi-institutional analysis, stimulant use in ADHD was moderately associated with increased URTI risk. These findings warrant consideration in risk-benefit discussions for ADHD treatment, particularly for patients with frequent infections or compromised immunity. Future prospective studies should explore dose-response effects, adherence, and biologic mediators to clarify causality and guide preventive strategies.

## Introduction

Attention-deficit/hyperactivity disorder (ADHD) is a common neurodevelopmental disorder, affecting approximately 8% of children and adolescents and 2.5% of adults globally [1,2]. Stimulant medications, such as methylphenidate and amphetamine derivatives, are first-line treatments and have demonstrated efficacy in reducing ADHD symptoms [3]. However, these medications are associated with a range of systemic side effects, and concerns have been raised about their potential immunomodulatory effects [4].

Upper respiratory tract infections (URTIs) are among the most common reasons for ambulatory care visits and school absenteeism in both children and adults [5]. The relationship between ADHD medications and susceptibility to URTIs has not been well studied. Stimulants can influence immune function via catecholaminergic activity and hypothalamic-pituitary-adrenal axis modulation, potentially impacting host susceptibility to infectious agents [6]. Furthermore, stimulant medications are well known for their potential to delay sleep onset and cause insomnia, especially when taken too late in the day [7]. This decrease in quality and quantity of sleep is another possible mechanism by which ADHD medications may influence immune function and possible susceptibility to URTIs [8].

Prior studies have found mixed evidence regarding how ADHD and ADHD medication use influence respiratory infectious disease risk. Chen et al. found ADHD to be associated with increased emergency department visits and hospitalizations for infectious diseases and that this risk may be mitigated with methylphenidate use [9]. A clinical trial found URTIs were more common in patients treated with methylphenidate compared to controls, and a population-based case-control study found that ADHD itself correlated with increased rates of childhood infectious diseases [10,11].

This study seeks to examine whether pharmacologic treatment of ADHD is associated with an increased risk of URTIs using data from the TriNetX research network, a large, federated database of real-world clinical encounters.

## Materials and methods

We performed a retrospective cohort study using the TriNetX database, aggregating de-identified patient data over 170 million electronic health records from 149 participating healthcare organizations globally. The TriNetX database provides a variety of information, including diagnoses, prescriptions, procedures, demographics, and laboratory values [12]. TriNetX data was accessed via connection through Drexel University for this study, spanning patient data from 2000 to 2025.

Two cohorts were identified utilizing the International Classification of Diseases, 10th Revision (ICD-10) codes as inclusion and exclusion criteria. Our study compared URTIs between cohorts. The control cohort was defined as having any of the following ICD-10 codes: “UMMLS:ICD10CM:F90.1 Attention-deficit hyperactivity disorder, predominantly hyperactive type”, “UMLS:ICD10CM:F90.2, Attention-deficit hyperactivity disorder, combined type”, or “UMLS:ICD10CM:F90.0, Attention-deficit hyperactivity disorder, predominantly inattentive type”. This query produced our control cohort with 1,798,001 patients. The comparison cohort, which we called the medication cohort, was defined by the following codes: “UMMLS:ICD10CM:F90.1 Attention-deficit hyperactivity disorder, predominantly hyperactive type”, “UMLS:ICD10CM:F90.2, Attention-deficit hyperactivity disorder, combined type”, or “UMLS:ICD10CM:F90.0, Attention-deficit hyperactivity disorder, predominantly inattentive type” and any of the medication codes for amphetamine, dextroamphetamine, lisdexamfetamine, methylphenidate, modafinil, or dexmethylphenidate. This query produced our medication cohort with 1,099,756 patients. For this study, our outcome of acute upper respiratory infection was identified with any of the ICD-10 codes “UMLS:ICD10CM:J00-J06”.

In the two-comparison cohort component of the study, measures of association and survival were assessed using the TriNetX platform. Risk difference and risk ratio were calculated to compare cohorts. Survival was extrapolated using a Kaplan-Meier survival analysis, followed by a log-rank test, and a Cox hazard ratio and proportionality test. The survival probability of the observed outcome at the end of the time window was calculated and compared. In this study, survival probability refers to the probability that a patient survives without experiencing the outcome of interest, so a higher proportion would indicate fewer patients in that population experienced an acute URTI. The log-rank test was used to assess significance (p < 0.05). Hazard ratios were calculated using the Cox proportional hazards model. The index event was defined using ICD-10 codes. For both cohorts, their respective index events were defined the same as their inclusion criteria for cohort generation. This analysis included outcomes that occurred in the time window that started one day after the first occurrence of the index event. Because of the limitations of the platform use, subgroup analyses were not performed.

## Results

In all, 2,897,757 patients with a diagnosis of ADHD were identified. These patients were categorized into the two aforementioned cohorts: the control cohort contained 1,798,001 patients with ADHD who were not treated with stimulant medications. The medication cohort contained 1,099,756 patients with ADHD who were treated with stimulant medications. We did not include antidepressants or antipsychotics in the cohort queries.

The control cohort had an average age of 20.6 years (SD ± 15), with 41.4% (n=744,446) being female, 56.18% (n=1,010,110) being male, and 2.42% (n=43,445) being of unknown sex. In regard to race, 67.96% (n=1,222,006) of the patients identified as White individuals, 10.32% (n=185,558) as Black individuals, 1.98% (n=35,647) as Asian individuals, 0.4% (n=7,229) as American Indian or Alaska Native individuals, and 0.27% (n=4,769) as Native Hawaiian or Other Pacific Islander individuals. In this cohort, 3.98% (n=71,501) of patients marked their race as Other, and 15.09% (n=271,291) of patients did not have their race documented (Table 1).

**Table 1:** Demographic characteristics by group

	Control group (n=1,798,001)	Medicated group (n=1,099,756)
Age, mean ± SD	20.6 ± 15	21.9 ± 15.4
Gender, n (%)		
Female	744,446 (41.4)	475,337 (43.22)
Male	1,010,110 (56.18)	601,546 (54.7)
Unknown	43,445 (2.42)	22,873 (2.08)
Race, n (%)		
White individuals	1,222,006 (67.96)	799,541 (72.7)
Black or African American individuals	185,558 (10.32)	110,365 (10.03)
Asian individuals	35,647 (1.98)	20,514 (1.87)
American Indian or Alaska Native individuals	7,229 (0.4)	4,641 (0.42)
Native Hawaiian or Other Pacific Islander individuals	4,769 (0.27)	2,286 (0.21)
Other	71,501 (3.98)	2,664 (5)
Unknown	271,291 (15.09)	121,398 (11.04)
Ethnicity, n (%)		
Hispanic or Latino	131,806 (7.33)	577,953 (7.09)
Not Hispanic or Latino	1,144,669 (63.66)	744,947 (67.74)
Unknown	521,526 (29.01)	276,856 (25.17)

Regarding the medication cohort, the average patient age was 21.9 years (SD ± 15.4), with 43.22% (n=475,337) being female, 54.7% (n=601,546) being male, and 2.08% (n=22,873) being of unknown sex. Regarding race, 72.7% (n=799,541) of patients were White individuals, 10.03% (n=110,365) were Black individuals, 1.87% (n=20,514) were Asian individuals, 0.42% (n=4,641) were American Indian or Alaska Native individuals, and 0.21% (n=2,286) were Native Hawaiian or Other Pacific Islander individuals. In this cohort, 3.73% (n=41,011) of patients marked their race as Other, and 11.04% (n=121,398) did not have their race documented (Table 1).

In the control cohort, URTIs were documented in 28.5% of patients (n=512,849). While in the medication cohort, URTIs were documented in 31.2% of patients (n=343,385). Stimulant use was associated with a significant increased risk of URTIs; the results indicated a risk difference of 0.027 (95% confidence interval (CI): 0.026-0.028, z=48.892, p<0.001), a risk ratio of 1.095 (95% CI: 1.091-1.099), and an odds ratio of 1.138 (95% CI: 1.132-1.144) (Tables 2, 3).

**Table 2:** Measures of association: cohort risk, risk ratio, and odds ratio CI: confidence interval

Control group risk	Medicated group risk	Risk ratio (95% CI)	Odds ratio (95% CI)
0.285	0.312	1.095 (1.091, 1.099)	1.138 (1.132, 1.144)

**Table 3:** Measures of association: risk difference CI: confidence interval

Risk difference (95% CI)	Z	p-value
0.027 (0.026, 0.028)	48.892	<0.001

**Figure 1:**
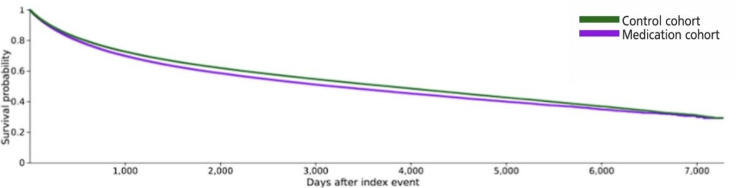
Kaplan-Meier analysis: survival curved

The results of the Kaplan-Meier survival analysis indicated that patients in the control cohort had a survival probability (i.e., chances of remaining URTI-free) of 29.29% whereas their counterparts in the medication cohort had a survival probability of 29.18%. The median survival period was 3757 days for the control cohort and 3176 days for the medication cohort (Figure 1). The difference between these survival curves was verified to be statistically significant with a log-rank test (χ²= 2285.024; p<0.001) (Table 4).

**Table 4:** Kaplan-Meier analysis: log rank test

χ^2^	df	p-value
2285.024	1	<0.001

Patients in the medicated cohort also had an 11.1% higher risk of developing a URTI compared to their control cohort counterparts. Hazards analysis yielded a hazard ratio of 1.111 (95% CI: 1.106-1.116, p<0.001) (Table 5).

**Table 5:** Kaplan-Meier analysis: hazard ratio and proportionality CI: confidence interval

Hazard ratio (95% CI)	χ^2^	df	p-value
1.111 (1.106, 1.116)	59.962	1	<0.001

## Discussion

Our analysis found that patients with ADHD who were prescribed stimulant medications had a statistically significantly higher incidence of URTIs compared to those who were not medicated. This association persisted after adjusting for potential confounders through propensity score matching.

Our findings add to a growing body of literature with mixed conclusions. For instance, Chen et al. reported that ADHD was associated with increased emergency department visits and hospitalizations for infectious diseases but suggested that this risk may be attenuated by methylphenidate use [9]. Conversely, a clinical trial reported that URTIs were more common in patients treated with methylphenidate, and another population-based case-control study found ADHD to be correlated with increased rates of childhood infectious diseases [10,11,13]. Our study aligns more closely with the latter, supporting an association between stimulant use and increased URTI risk. These differences in findings may reflect variation in study design, populations studied, or definitions of outcomes, highlighting the need for further targeted research in this area.

There are plausible biological explanations for this finding. Stimulants increase sympathetic nervous system activity, which can alter immune function by suppressing lymphocyte proliferation and shifting cytokine profiles [6,14]. This may impair mucosal immunity, thereby increasing susceptibility to respiratory pathogens. Alternatively, increased social exposure resulting from improved functional behavior on medication (e.g., greater school or work attendance) could elevate infection risk.

In addition to the potential for direct immune modulation via increased sympathetic nervous system activity associated with stimulant use, several other plausible mechanisms may contribute to this observed increased risk of URTIs in the medication cohort. Stimulant medications, including amphetamines and methylphenidate, are known to cause insomnia and reduced sleep quality, both of which can impair immune function. Even short-term sleep deprivation has been shown to reduce natural killer cell activity, impair T-cell function, and increase susceptibility to viral infections such as the common cold [15,16]. Chronic poor sleep is associated with systemic inflammation and blunted immune responses to pathogens, which may heighten the risk of respiratory tract infections [17]. Thus, stimulant-induced sleep disturbances may serve as an indirect pathway by which these medications increase susceptibility to infection.

Another possible mechanism is through the induction of dry mouth, a well-documented side effect of stimulant medications [18]. Saliva plays an essential role in maintaining oral and upper airway mucosal health by providing antimicrobial peptides, immunoglobulins, and mechanical clearance of pathogens [19]. A reduction in salivary flow can compromise this first-line defense, allowing increased microbial colonization of the oropharyngeal and upper respiratory tract, thereby potentially increasing the risk of URTIs. Furthermore, dry mucosa may impair mucociliary clearance, further facilitating infection [20]. These risks related to stimulant medications have possible associations with COVID-19 infections and influenza, which may be related to similarities in mode of transmission and host defense [21].

A major strength of our study is the use of the TriNetX database, which provides access to a large, diverse cohort of patients across multiple healthcare organizations. This enhances the external validity of our findings and increases the power of our statistical analyses. Additionally, the use of standardized diagnostic codes ensures consistency in cohort identification and outcome measurement. By focusing on a clearly defined clinical outcome (URTI) and a well-characterized exposure (ADHD stimulant medication use), we reduce the risk of misclassification bias.

However, several limitations should be acknowledged. This study used observational data, which inherently limits causal inference. Adherence to prescribed stimulant medications was not directly measured. The retrospective nature of the study also limits control over potential confounding variables such as socioeconomic status, environmental exposure, comorbid conditions, and over-the-counter medication use. Additionally, we were unable to assess the dose or duration of stimulant medication used, nor could we distinguish between URTI severity or microbiological etiology. Another limitation is that stimulant use may be underreported if patients did not fill prescriptions or if medications were prescribed outside the reporting institutions. Lastly, social factors, such as increased social engagement or differing health-seeking behaviors in medicated versus non-medicated individuals, could partially explain the observed association. The proposed mechanisms of association between ADHD and URTI are not fully determined in this study.

Future research should address these limitations through prospective cohort studies with more granular data on medication adherence, dosing, and duration of stimulant use. Studies incorporating biomarkers of immune function or sleep quality could help elucidate the mechanistic pathways underlying the observed association. Additionally, examining whether certain subgroups (e.g., children vs. adults) are at higher risk could guide personalized risk mitigation strategies. Investigating whether behavioral or pharmacological interventions (e.g., sleep hygiene measures, saliva substitutes) can reduce URTI risk in this population may also prove valuable.

## Conclusions

In this large retrospective cohort study, we found that ADHD patients treated with stimulant medications had a modest but statistically significant increased risk of developing URTIs compared to their non-medicated counterparts. While several plausible biological mechanisms may explain this association, further research is warranted to confirm these findings and explore strategies to minimize infection risk in this population. These findings highlight the importance of comprehensive risk-benefit discussions when initiating ADHD pharmacotherapy, particularly in individuals with frequent infections or other risk factors for impaired immunity.
